# Editorial: Transcriptional Regulation in Metabolism and Immunology

**DOI:** 10.3389/fgene.2022.845697

**Published:** 2022-02-02

**Authors:** Chunjie Jiang, Shibiao Wan, Peng Hu, Yongsheng Li, Shengli Li

**Affiliations:** ^1^ Department of Medicine, Division of Diabetes, Endocrinology and Metabolism, Baylor College of Medicine, Houston, TX, United States; ^2^ Center for Applied Bioinformatics, St. Jude Children’s Research Hospital, Memphis, TN, United States; ^3^ College of Fisheries and Life Science, Shanghai Ocean University, Shanghai, China; ^4^ Key Laboratory of Tropical Translational Medicine of Ministry of Education, College of Biomedical Information and Engineering, Hainan Medical University, Haikou, China; ^5^ Precision Research Center for Refractory Diseases, Institute for Clinical Research, Shanghai General Hospital, Shanghai Jiao Tong University School of Medicine, Shanghai, China

**Keywords:** transcriptional regulation, next-generation sequencing, transcription factor, metabolism, immunology

The regulation of transcription that converts DNA to RNA is a vital process in all living organisms to orchestrate gene activities (Weingarten-Gabbay and Segal, 2014; Cramer, 2019). Transcription factors (TFs) are important factors to orchestrate transcription by binding to specific DNA sequences to activate or repress wide repertoires of downstream target genes that control a wide variety of biological processes (Spitz and Furlong, 2012; Lambert et al., 2018), including metabolic and immune systems. A large number of TFs that play critical roles in regulating transcription in the metabolic and immune systems have been investigated and much has been learned about their mechanisms (Mansueto et al., 2017; Hosokawa and Rothenberg, 2021).

Metabolic homeostasis needs fine tuning to adapt to environmental stimuli, which largely depends on transcriptional-level regulation (Mouchiroud et al., 2014). Maintenance of energy homeostasis is critical in all cells, which is mainly perceived and regulated by the highly conserved AMP-activated protein kinase (AMPK) (Garcia and Shaw, 2017). AMPK has been shown to phosphorylate specific transcription factors, such as FOXO transcription factors, to restore energy balance and reprogram many metabolic progresses, including the metabolism of glucose, lipid, mTOR, and proteins. Nonalcoholic fatty liver disease (NAFLD) is the most prevalent liver disease worldwide, which may progress to fatal cirrhosis or hepatocellular carcinoma (Foulds et al., 2017). Exposure to endocrine-disrupting chemicals (EDCs) may increase the susceptibility to the development of NAFLD. Imbalance of hepatic lipid homeostasis may lead to the initiation and development of NAFLD. EDCs can recruit co-regulator proteins by physically binding nuclear receptors (NRs), and modulate the transcription of genes involved in hepatic lipid homeostasis.

Trigger of required immune response demands fine transcriptional regulation in cells of the immune system (Roy, 2019). Wu *et al.* applied single-cell RNA sequencing to investigate IL-4-induced I transcription in B cell differentiation (Wu et al., 2017). Their analysis revealed that the early transcription of Iε could induce class switching to IgE. Thus, the transcription regulation of Iε directs the early choice of IgE. In addition, various noncoding RNAs have been found to participate in the regulation of immune processes and immune cells, including circular RNAs and long noncoding RNAs (Hu W. et al., 2021; Fang et al., 2021).

This Research Topic is dedicated to publishing studies revealing the mechanisms of transcriptional regulation in metabolic and/or immune systems based on the data sets from next-generation sequencing and other state-of-art technologies, which will shed light on the deeper understanding of the underlying mechanisms. A total of 19 articles are included in this Research Topic.

Four papers contributed to the transcriptional regulation in metabolic system. Zhang *et al.* revealed five metabolism pathway-related circRNAs in prostate cancer (Zhang et al.). Cheng *et al.* found that alterations in lipid metabolism pathway are associated with prognosis of non-small-cell lung cancer patients that were treated with immune checkpoint inhibitors (Cheng et al.). One research performed systematic analysis of nuclear-encoded mitochondrial genes in hypertrophic cardiomyopathy, including the regulation of transcription factors (Tan et al.). Liu *et al.* examined the dysregulation of immune and metabolism-related RNAs in uterine corpus endometrial carcinoma (Liu and Qiu).

For the transcriptional regulation in immune system, two articles contributed to the transcriptional dysregulation in immune cells and their roles as biomarkers in diseases, including macrophage M2 cells (Wang et al.) and neutrophils (Qiu et al.). Several articles identified immune-related prognostic markers in human complex diseases, including stromal-immune scores (Liu et al.), lncRNAs (Wang et al.; Pang et al.; Zhao et al.), immune-related genes (Hu et al.; He et al.; Li et al.; Xu et al.), and transcriptional regulation factors (He et al.; Zhang et al.; Chen et al.).

In addition, the Research Topic also included two methodology articles, one is about a deep learning classifier for determining disease immune subtypes and related immunosuppression genes (Ning et al.), and the other is the comparisons of dimensionality reduction methods in single-cell transcriptomics data (Xiang et al.).

In conclusion, recent studies have precisely highlighted dysregulated TFs in specific contexts by adopting high throughput sequencing and other state-of-the-art technologies. These studies largely extended our current knowledge of the complexity of gene regulation circuitry in metabolism and immunology, and will facilitate further advancement.
